# The Association of Body Mass Index With Dietary Habits, Screen Time, and Physical Activity Among School Children in Sri Vijaya Puram, Andaman and Nicobar Islands, India

**DOI:** 10.7759/cureus.92599

**Published:** 2025-09-18

**Authors:** Aanchal Anand, Samar Hossain, Ajay Raj Sethuraman, Akansha Tomar, D Yashika

**Affiliations:** 1 Community Medicine, Andaman and Nicobar Islands Institute of Medical Sciences, Sri Vijaya Puram, IND

**Keywords:** body mass index, fast food, physical activity, school children, screen time

## Abstract

Background and objective

In India, the rising trend of childhood overweight and obesity is observed not only in urban but also in semi-urban and remote settings. Sedentary behavior, unhealthy diets, and screen overexposure are the major contributors to this phenomenon. However, limited evidence exists from geographically underrepresented locations such as Sri Vijaya Puram, Andaman and Nicobar Islands. This study aimed to assess the association of BMI with dietary habits, screen time, and physical activity among school-going children in Sri Vijaya Puram.

Materials and methods

This was a cross-sectional study conducted during a one-day health camp among 259 school children (Classes 1-10) in a co-educational school in Sri Vijaya Puram. Anthropometric measurements were recorded using standardized tools. A pre-tested structured questionnaire was employed to assess dietary patterns, screen time, fast food and sugary beverage consumption, and physical activity. Data were analyzed using SPSS Statistics version 27 (IBM Corp., Armonk, NY).

Results

Higher BMI was significantly associated with increased screen time, frequent intake of fast foods/sugary beverages, and inadequate physical activity. Notable behavioral variation was seen between weekdays and weekends.

Conclusions

Modifiable lifestyle behaviors strongly influence BMI. School-based health promotion and parental engagement are essential to curb childhood obesity.

## Introduction

Childhood obesity has become a global health crisis, with the World Health Organization (WHO) reporting a staggering number of over 340 million children and adolescents (aged 5-19 years) as overweight or obese in 2016 [1]. This epidemic is no longer limited to high-income nations; its prevalence is rising rapidly in low- and middle-income countries as well, including India. This phenomenon is largely attributed to a global nutrition and lifestyle transition that has led to a significant shift in dietary patterns towards energy-dense, processed foods and a simultaneous decrease in physical activity due to sedentary behaviors and increased screen time [2]. The consequences of childhood obesity are far-reaching, extending beyond immediate health issues to an increased risk of developing chronic diseases such as type 2 diabetes, hypertension, and cardiovascular disease later in life.

In India, the dual burden of malnutrition coexisting with rising obesity presents a unique public health challenge, especially in urban and semi-urban areas [3]. BMI, a widely accepted and accessible screening tool, is crucial for assessing nutritional status and identifying children at risk of future health complications [4]. A substantial body of evidence has established a strong association between modifiable lifestyle behaviors and elevated BMI in children [5,6]. These key behaviors include excessive screen time, frequent consumption of fast food and sugar-sweetened beverages, and insufficient physical activity. While national surveys offer valuable macro-level insights, there is a critical paucity of local, community-specific data that can inform targeted interventions. This is particularly true for rapidly developing urban settings like Sri Vijaya Puram, Andaman and Nicobar Islands, which are experiencing accelerated lifestyle changes driven by urbanization and digital technology exposure.

Therefore, this study was undertaken to address this specific knowledge gap by investigating the association between key lifestyle behaviors - namely dietary habits, screen time, and physical activity - and the BMI status of school children aged 6-16 years in Sri Vijaya Puram. By providing a localized evidence base, this research aims to offer actionable insights for schools and community-based programs. The findings are intended to support the development of effective, evidence-informed strategies to promote healthier habits and prevent childhood obesity in this specific population at an early stage. We believe that this is a critical step towards mitigating the long-term health and economic burdens associated with this growing epidemic.

## Materials and methods

Study design and setting

A cross-sectional study was conducted during a one-day health camp in December 2024 at RGT Public School, a co-educational government school located in the urban area of Sri Vijaya Puram, Andaman and Nicobar Islands. Formal written permission was obtained from the Principal of the school prior to conducting the camp.

Sample size

The minimum required sample size was calculated as 194 participants based on the global prevalence of overweight among children and adolescents (14.8%) reported by Zhang et al [7] with an absolute allowable error of 5% and 95% confidence level (Z = 1.96) using the formula:

n = Z² × p × (1-p) / d² = (1.96² × 0.148 × 0.852) / 0.05² = 194

Enrollment approach

Our enrollment approach utilized a universal sampling method, whereby all 259 students present in the school on the day of the health camp were included in the study after verifying that each individual met our predetermined inclusion criteria. This enrollment exceeded the minimum sample requirement (n = 194), enhancing statistical power while maintaining uniform protocols. Participants were distributed across classes as follows: Class 1 (n = 37), Class 2 (n = 24), Class 3 (n = 24), Class 4 (n = 23), Class 5 (n = 40), Class 6 (n = 28), Class 7 (n = 22), Class 8 (n = 30), Class 9 (n = 14), and Class 10 (n = 17).

Inclusion criteria

Students aged 6-16 years provided assent, with parental consent obtained through class teachers. All participants met predefined inclusion criteria and underwent identical assessments. A total of 350 students were enrolled in the RGT public school from Std 1 to 10thas per school records. Absentees (n = 91) were excluded. All the students present in the school on the day of the health camp received equal health services, even if they were not a part of the study.

Final cohort and ethical approval

The study enrolled 259 participants (162 boys [62.5%], 97 girls [37.5%]), ensuring enhanced power to detect associations while reflecting the actual population attending the camp. Before initiating the study, ethical approval was obtained from the Institutional Ethics Committee of ANIIMS (approval number: ANIIMS/IEC/2024/77, dated December 21, 2024), along with formal permission from RGT Public School authorities. 

Participant enrollment and data collection

Only students providing assent (with parental consent facilitated through class teachers) were enrolled. Participants completed a structured bilingual questionnaire (English/local language) capturing self-reported dietary habits (fast food/sugary beverage frequency), screen time (weekday/weekend duration), and physical activity patterns (type/frequency/duration). This instrument was developed through a literature review and pre-tested for clarity, validity, and reliability in a pilot student sample.

Anthropometric classification

Nutritional status was classified using WHO BMI-for-age standards: underweight (≤2 standard deviation [SD]), normal (≥ -2 SD to ≤ +1 SD), and overweight (> +1 SD; combined overweight/obesity).

Data analysis

The data underwent double-entry verification and refinement in Microsoft Excel before analysis. Statistical analyses were performed using SPSS Statistics version 27 (IBM Corp., Armonk, NY), with continuous variables expressed as mean ± standard deviation (SD) and categorical variables as proportions. Associations between variables were assessed using chi-square tests, with statistical significance set at p<0.05.

## Results

Among the 259 school children included in the study, the largest age group was 9-11 years, comprising 36% of the participants, followed by six to eight years (30.1%) and 12-14 years (27.4%). Only 6.5% of students fell into the 15-16-year age bracket. A clear male predominance was noted, with 62.5% boys and 37.5% girls (Table 1).

**Table 1:** Sociodemographic characteristics of the study participants BMI: body mass index

Sociodemographic characteristics		
Variable	Frequency	%
Age group, years		
6-8	78	30.1
9-11	93	36
12-14	71	27.4
15-16	17	6.5
Gender		
Male	162	62.5
Female	97	37.5
BMI		
Underweight	75	28.9
Normal	81	31.2
Overweight	103	39.9

Assessment of nutritional status revealed that 39.9% of the children were overweight, while 28.9% were underweight. Only 31.2% had a normal BMI, highlighting the coexistence of both undernutrition and overnutrition: a concerning trend indicative of a dual burden of malnutrition in the school-aged population.

As shown in Table 2, a large proportion of participants (61.7%) reported occasional consumption of fast food, while 21.2% consumed it daily, indicating a notable presence of unhealthy dietary habits. Similarly, 83% of students occasionally consumed sugary beverages while 4.2% reported daily intake, reflecting increased exposure to sugar-laden drinks. In terms of screen time, a relatively healthy pattern was noted during weekdays, with 75.6% of children limiting screen exposure to less than two hours. However, this trend reversed on weekends, where only 30.8% maintained <2 hours of screen time, and 23.3% reported more than four hours of screen use, indicating significantly higher sedentary behavior during weekends.

**Table 2:** Distribution of study participants by lifestyle choices

Lifestyle choices		
Variable	Frequency	%
Fast food consumption		
Daily	55	21.2
Occasionally	160	61.7
Rarely	29	11.1
Never	15	6
Soft drink consumption		
Daily	11	4.2
Occasionally	215	83
Rarely	32	12.3
Never	1	0.5
Screen time (weekdays), hours		
<2	196	75.6
2-4	45	17.5
>4	18	6.9
Screen time (weekends), hours		
<2	80	30.8
2-4	119	45.9
>4	60	23.3

Table 3 highlights the most frequently observed health conditions among the study participants. Impacted earwax was the most prevalent, affecting 106 children (41%), followed by dental caries in 62 children (23.9%) and reduced visual acuity in 46 (17.8%). Male students showed a higher prevalence of dermatological conditions (22 males vs. seven females) as well as vision-related issues. Although less common, cases of tonsillitis and deviated nasal septum (DNS) were also identified during routine health assessments.

**Table 3:** Genderwise distribution of common morbidities among school children DNS: deviated nasal septum

Common morbidities		Male	Female	Total
Impacted earwax		64	42	106
Tonsillitis	Grade I	5	2	7
	Grade II	1	2	3
Reduced visual acuity		32	14	46
Dermatological conditions		22	7	29
Dental caries		39	23	62
DNS		2	0	2
Others		7	5	12

As summarized in Table 4, a significant association was observed between BMI and age group (χ² = 407.85, p<0.001). All children in the 9-11 age group who were underweight accounted for 100% of the underweight category, while all children in the six-eight-year group were overweight. The 12-14 age group predominantly had normal BMI. No statistically significant association was found between gender and BMI (χ² = 5.32, p = 0.070), suggesting that BMI distribution was independent of gender.

**Table 4:** Association between BMI and sociodemographic and lifestyle variables BMI: body mass index

BMI and sociodemographic and lifestyle variables												
	Underweight		Normal			Overweight	Total		X^2^		P-value	
Age group, years												
6-8	0 (0%)		0 (0%)			78 (75.7%)	78 (30.1%)					
9-11	75 (100%)		0 (0%)			18 (17.4%)	93 (35.9%)		407.85		<0.001	
12-14	0 (0%)		71 (87.6%)			0 (0%)	71 (27.4%)					
15-16	0 (0%)		10 (12.4%)			7 (6.9%)	17 (6.6%)					
Gender												
Male	55 (73.5%)		48 (59.2%)			59 (57.2%)	162 (62.5%)		5.32		0.070	
Female	20 (26.5%)		33 (40.8%)			44 (42.7%)	97 (37.5%)					
Soft drink consumption												
Daily	36 (48%)		12 (14.8%)			34 (33%)	82 (31.6%)				<0.001	
Occasionally	20 (26.6%)		65 (80.2%)			47 (45.8%)	132 (50.9%)		47.99			
Rarely	11 (14.6%)		3 (3.7%)			11 (10.6%)	25 (9.6%)					
Never	8 (10.8%)		1 (1.3%)			11 (10.6%)	20 (7.9%)					
Fast food consumption												
Daily	5 (6.6%)		4 (4.9%)			46 (44.6%)	55 (21.4%)					
Occasionally	66 (88%)		62 (76.5%)			32 (31%)	160 (61.7%)		83.31		<0.001	
Rarely	1 (1.4%)		9 (11.1%)			19 (18.5%)	29 (11.1%)					
Never	3 (4%)		6 (7.5%)			6 (5.9%)	15 (5.8%)					
Screen time (weekdays), hours												
<2	11 (14.6%)		21 (25.9%)			35 (33.9%)	67 (25.8%)		13.67			
2-4	51 (68%)		42 (51.8%)			42 (40.7%)	135 (52.1%)				0.0084	
>4	13 (17.4%)		18 (22.3%)			26 (25.4%)	57 (22.1%)					
Screen time (weekends), hours												
<2	7 (9.3%)		17 (20.9%)			36 (34.9%)	60 (23.1%)				<0.001	
2-4	45 (60%)		53 (65.4%)			53 (51.4%)	151 (58.3%)					
>4	23 (30.7%)		11 (13.7%)			14 (13.7%)	48 (18.6%)		22.49			

Soft drink consumption was significantly associated with BMI (χ² = 47.99, p<0.001). Children who consumed sugary beverages daily had higher rates of both overweight and underweight compared to those who drank them rarely or never. A highly significant association was noted between fast food consumption and BMI (χ² = 83.31, p<0.001). Daily fast food consumers predominantly belonged to the overweight category (44.6%). Screen time on weekdays showed a significant association with BMI (χ² = 13.67, p = 0.0084). Overweight status was more common among those with prolonged screen time (>4 hours). Similarly, weekend screen time also had a statistically significant association (χ² = 22.49, p = 0.0002), with a higher proportion of overweight children reporting >4 hours of screen exposure.

These findings highlight age, fast food consumption, soft drink intake, and screen time as major contributing factors to abnormal BMI distribution among children.

## Discussion

The present study reveals a dual burden of malnutrition among school-aged children in the Andaman and Nicobar Islands, with 28.9% classified as underweight and 39.9% as overweight. This coexistence of undernutrition and overnutrition reflects a nutritional transition observed in various parts of India [8]. For instance, a study analyzing data from 2006 to 2021 indicated a decrease in underweight prevalence by 10.3 percentage points, while overweight prevalence increased by 1.9 percentage points during the same period [8].

The significant association between age and BMI in our study, with younger children (six to eight years) predominantly overweight and older children (9-11 years) more often underweight, suggests age-specific nutritional challenges. This pattern aligns with findings from a study by Blakenship et al. in South Asia, where a triple burden of malnutrition - including undernutrition, overnutrition, and micronutrient deficiencies - has been documented among school-aged children [9]. The early onset of overweight in younger children may result from increased exposure to high-calorie diets and sedentary lifestyles, while undernutrition in older children could be linked to socioeconomic factors and inadequate dietary intake.

Dietary habits, particularly fast food and soft drink consumption, were significantly associated with BMI. Children who consume fast food daily exhibited higher rates of overweight. This finding is consistent with research done by Mohammadbeigi et al. in 2018, indicating that increased fast food intake and sweets consumption are statistically associated with adolescent obesity [10]. The aggressive marketing of ultra-processed foods and beverages, often misleadingly promoted as beneficial, contributes to unhealthy eating behaviors and subsequent weight gain. Screen time was another critical factor linked to BMI, with prolonged exposure on both weekdays and weekends correlating with higher overweight prevalence. A study conducted in Pune, India, reported that 83.2% of secondary school children had excess screen time, which was significantly associated with inadequate sleep and other lifestyle factors. Increased screen time contributes to sedentary behavior and may displace physical activity, exacerbating the risk of obesity [11].

The lack of a significant association between gender and BMI in our study suggests that both boys and girls are equally exposed to unhealthy lifestyle environments within the school setting. However, other studies have reported gender differences in obesity prevalence, emphasizing the need for further research to explore potential sociocultural and behavioral factors influencing these outcomes [12]. The high prevalence of dental caries (24%) and reduced visual acuity (18%) among participants underscores the importance of integrating oral and visual health interventions within school health programs. These conditions can adversely affect academic performance and quality of life, highlighting the need for regular screenings and preventive measures [13].

Our findings emphasize the necessity for comprehensive, multifaceted interventions targeting dietary habits, physical activity, and screen time to address the dual burden of malnutrition. School-based programs promoting nutrition education, healthy eating behaviors, and active lifestyles are essential. Additionally, policies regulating the marketing of unhealthy foods and beverages to children, as recommended by the World Health Organization, could help mitigate exposure to sedentary and high-calorie environments. In conclusion, the study highlights the complex interplay of socio-demographic and lifestyle factors influencing nutritional status among school children in the Andaman & Nicobar Islands. Addressing these challenges requires coordinated efforts involving schools, families, policymakers, and the broader community to create supportive environments that foster healthy growth and development.

Strengths of the study

One of the primary strengths of this study lies in its comprehensive inclusion of the entire accessible population from a school setting, resulting in a relatively large and diverse sample size of 259 children across various age groups (6-16 years). This inclusive sampling approach enhances the representativeness of the findings. The use of a structured, pre-tested questionnaire in both English and the local language ensured cultural and linguistic relevance, thereby improving the accuracy of self-reported data. Anthropometric measurements were obtained using standard protocols and calibrated instruments, which strengthens the internal validity of BMI categorization. The study also applied robust statistical methods, including Chi-square analysis, to identify associations between BMI and key socio-demographic and lifestyle variables. Furthermore, this study is among the few from the Andaman & Nicobar Islands to explore lifestyle-related obesity risk factors, thus contributing novel regional data to the national discourse on childhood nutrition.

Limitations of the study

As a cross-sectional study conducted in a single school on a single day, the findings may not be generalizable beyond this setting. Self-reported data may be subject to recall bias, especially among younger students. Important factors such as socioeconomic status and parental influence were not assessed. The absence of longitudinal data limits causal inference. Nonetheless, the study offers valuable baseline insights to inform school-based health interventions.

## Conclusions

This study offers a glimpse into the shifting health patterns of school-going children in a rapidly urbanizing part of the Andaman and Nicobar Islands. What clearly stands out is the growing influence of lifestyle choices - frequent fast food consumption, sugary drinks, and extended screen time - all of which show a strong link with rising BMI levels. At the same time, the presence of undernutrition alongside overweight reflects a double challenge that cannot be overlooked. These findings highlight the importance of nurturing healthy habits early in life. Schools, parents, and local health authorities should work together in promoting better nutrition and more active daily routines. Small, sustained changes - like healthier school meals, encouraging playtime, and limiting screen exposure - can go a long way in supporting children’s well-being and preventing long-term health issues.

## Appendices

SCHOOL HEALTH, LIFESTYLE, AND NUTRITION ASSESSMENT For School Children in Sri Vijaya Puram, Andaman & Nicobar Islands

SECTION A: CHILD AND FAMILY DEMOGRAPHICS

(To be completed by Parent/Guardian)

Question

Response Options

A1. Child's Name (Optional):

_______________________

A2. Age:

______ Years ______ Months

A3. Gender:

□ Male □ Female □ Other

A4. Standard/Grade:

_______________________

A5. Date of Birth (DD/MM/YYYY):

__ / __ / ________

A6. Type of Family:

□ Nuclear □ Joint □ Extended □ Single Parent

A7. Number of Family Members Living Together:

______

A8. Number of Siblings:

______

A9. Child's Birth Order:

______ (e.g., 1st, 2nd)

A10. Residential Area:

□ Urban □ Rural □ Tribal Settlement

A11. Primary Caregiver 1 - Relationship:

□ Mother □ Father □ Grandparent □ Other: ______

Education Level:

□ No School □ Primary □ Secondary □ Higher Secondary □ Graduate □ Post Graduate

Occupation:

□ Govt. Job □ Private □ Self-Employed □ Agriculture/Fishery □ Labourer □ Homemaker □ Unemployed □ Retired

A12. Primary Caregiver 2 (if applicable) - Relationship:

□ Mother □ Father □ Grandparent □ Other: ______

Education Level:

[Same as A11]

Occupation:

[Same as A11]

A13. Approximate Monthly Family Income (₹):

□ <5,000 □ 5,000-10,000 □ 10,001-20,000 □ 20,001-30,000 □ 30,001-50,000 □ >50,000

A14. Main Source of Drinking Water:

□ Piped at Home □ Public Tap □ Tube Well/Borehole □ Well □ Rainwater □ Other: ______

A15. Type of Toilet Facility:

□ Flush Toilet □ Pit Latrine □ Public Latrine □ Open Defecation □ Other: ______

A16. Main Cooking Fuel:

□ LPG □ Firewood □ Kerosene □ Electricity □ Other: ______

SECTION B: DIETARY HABITS & FOOD ENVIRONMENT

*(Parent/Guardian for Ages 6-11; Self-report for Ages 12-16)*

Question

Response Options

B1. Did your child eat breakfast yesterday?

□ Yes □ No → If Yes: Time: ____ AM

Foods: _________________________

B2. Morning Snack (e.g., school tiffin):

□ Yes □ No → If Yes: Foods: _________________________

B3. Lunch Location & Foods:

Location: □ Home □ School Meal □ Packed □ Bought Outside → Foods: ______________

B4. Evening Snack:

□ Yes □ No → If Yes: Foods: _________________________

B5. Dinner Time & Foods:

Time: ____ PM

Foods: _________________________

B6. Food After Dinner?

□ Yes (Specify: _________) □ No

Food Frequency (Last Week):

How often did your child consume these?

Food Item

Daily

4-6x/week

2-3x/week

Once

Never

a) Fresh Fruits

□

□

□

□

□

b) Cooked Vegetables

□

□

□

□

□

c) Green Leafy Vegetables

□

□

□

□

□

d) Milk/Curd/Buttermilk

□

□

□

□

□

e) Pulses/Legumes (Dal)

□

□

□

□

□

f) Eggs

□

□

□

□

□

g) Fish/Meat/Chicken

□

□

□

□

□

h) Fast Food (Pizza, Burgers, Noodles)

□

□

□

□

□

i) Fried Snacks (Chips, Pakoras)

□

□

□

□

□

j) Sweets (Chocolates, Ice Cream)

□

□

□

□

□

k) Packaged Bakery (Biscuits, Cakes)

□

□

□

□

□

l) Sugary Drinks (Soda, Packed Juices)

□

□

□

□

□

B7. Food Environment Questions

Response Options

Who decides child's food?

□ Child □ Mother □ Father □ Grandparent □ Jointly

Eats meals while watching screens?

□ Always □ Often □ Sometimes □ Rarely □ Never

Family meals together?

□ Daily □ Most Days □ Weekends □ Rarely □ Never

Packs homemade school food?

□ Daily □ Most Days □ Sometimes □ Rarely □ Never

Buys snacks from school canteen?

□ Daily □ Weekly □ Monthly □ Rarely □ Never

Sees ads for fast food?

□ Very Often □ Often □ Sometimes □ Rarely □ Never

SECTION C: SCREEN TIME & SEDENTARY BEHAVIOR

*(Parent report for Ages 6-11; Self-report for Ages 12-16)*

Screen Time (Last School Week):

Activity

Avg. Time/Day (Weekdays)

Device Used (✓)

TV/Videos

______ : ______

□ Phone □ TV □ Tablet □ Laptop

Video Games

______ : ______

□ Phone □ TV □ Tablet □ Laptop

Social Media/Internet

______ : ______

□ Phone □ TV □ Tablet □ Laptop

TOTAL (Weekdays)

______ : ______

Screen Time (Last Weekend):

Activity

Avg. Time/Day (Weekend)

Device Used (✓)

TV/Videos

______ : ______

□ Phone □ TV □ Tablet □ Laptop

Video Games

______ : ______

□ Phone □ TV □ Tablet □ Laptop

Social Media/Internet

______ : ______

□ Phone □ TV □ Tablet □ Laptop

TOTAL (Weekend)

______ : ______

| C1. Uses screens while eating? | □ Often □ Sometimes □ Rarely □ Never |
 

| C2. Homework/Reading (hrs/day): | ______ |
 

| C3. Sitting/Resting (hrs/day): | ______ |

SECTION D: PHYSICAL ACTIVITY & SLEEP

Question

Response Options

D1. School Transport Method:

□ Walk □ Bicycle □ Bus □ Car/Bike □ Auto □ Boat

If Walk/Bike: Time (one way):

______ minutes

D2. Days with PE Class (last week):

______ days

D3. Recess Activity Level:

□ Very Active □ Somewhat Active □ Not Active

D4. Organized Sports?

□ Yes □ No → If Yes: Sport: _________ Days/week: ____ Time/session: ____ min

D5. Active Play (e.g., running):

Days/week: ____

Duration/day: ____ hrs ____ min

D6. School-Night Bedtime:

______ PM

D7. School-Night Wake-up Time:

______ AM

D8. Weekend Bedtime:

______ PM

D9. Screens Before Bed?

□ Often □ Sometimes □ Rarely □ Never

SECTION E: HEALTH STATUS & PERCEPTIONS

For Parents:

Response Options

E1. Child's Weight Perception:

□ Very Underweight □ Slightly □ About Right □ Slightly Overweight □ Very Overweight

E2. Child's Overall Health:

□ Excellent □ Very Good □ Good □ Fair □ Poor

E3. Dental Problems?

□ Yes □ No □ Don’t Know

E4. Vision Problems?

□ Yes □ No □ Don’t Know

E5. Chronic Conditions?

□ Yes (Specify: _________) □ No

For Children (Age 10+):

E6. Body Size Perception:

□ Too Thin □ Thin □ About Right □ Heavy □ Too Heavy

E7. Health Satisfaction:

□ Very Happy □ Happy □ Neutral □ Unhappy □ Very Unhappy

SECTION F: RESEARCHER MEASUREMENTS

Anthropometry

Measurement

Height (cm):

______ cm

Weight (kg):

______ kg

BMI (kg/m²):

______

BMI Category:

□ Underweight □ Normal □ Overweight □ Obese

Clinical Observations

Visible Dental Caries?

□ Yes □ No □ Cannot Assess

Wears Glasses?

□ Yes □ No

Other Health Notes:

_________________________
